# Psychogenic Nonepileptic Seizures after Head Injury: A Case Report

**DOI:** 10.1155/2009/712813

**Published:** 2009-10-25

**Authors:** Laura Scévola, Luciana D'Alessio, Dario Saferstein, Estela Centurión, Damián Consalvo, Silvia Kochen

**Affiliations:** ^1^Psychiatry Division, Ramos Mejía Hospital, Buenos Aires 1405, Argentina; ^2^Epilepsy Center, Ramos Mejía Hospital, Buenos Aires, Argentina

## Abstract

Psychogenic nonepileptic seizures (PNESs) are diagnosed when disruptive changes in behaviour, thinking, or emotion resemble epileptic seizures (ESs), but no paroxysmal discharges are seen on electroencephalogram (EEG) and do not originate from another medical illness. The gold standard for PNES diagnosis is video electroencephalogram (Video-EEG). PNESs are defined by modern psychiatry as conversion and dissociative disorders but these disorders may coexist with many others psychiatric disorders, including depression, posttraumatic stress disorder, and personality disorders. It is well known that epileptic seizures are a frequent and well-studied complication of traumatic head injury (THI). However, THI may also generate psychic symptoms including PNES. In this paper we describe a patient who developed PNES after THI in a bus accident and received a diagnosis of refractory epilepsy for 24 years until she underwent Video-EEG.

## 1. Introduction

Psychogenic nonepileptic seizures (PNESs), also known as pseudoseizures, are diagnosed when disruptive changes in behaviour, thinking, or emotion resemble epileptic seizures (ES), but no paroxysmal discharges are seen on electroencephalogram (EEG) and do not originate from another medical illness [1–3].

The diagnosis of PNES accounts for 10 to 40% of all patients referred for Video-EEG in specialized epilepsy centers [2, 4, 5]. Diagnosis of PNES is suspected by anamnesis, physical examination, ictal semiology, and personal and psychiatric history, but the gold standard for diagnosis is video electroencephalogram (Video-EEG). This technique results in a definitive diagnosis in almost 90% of patients [2, 6–12].

PNESs are defined by modern psychiatry as conversion disorder which is classified as one of the somatoform disorders in DSM IV [10] and is considered a subtype of dissociative disorder in ICD 10 [13]. Conversion disorder is a mental disorder whose central feature is the appearance of symptoms affecting the patient's senses or voluntary movements that suggest a neurological or general medical disease. Dissociative disorder is defined as a disruption in the usually integrated functions of consciousness, memory, identity, or perception of the environment [10]. Furthermore PNES may coexist with many psychiatric disorders, including depression, posttraumatic stress disorder, and personality disorders [2–4, 10, 14–16].

Patients with PNES report traumatic events more frequently than the general population. Several studies showed that the risk of PNES increases when there are antecedents of sexual abuse, physical abuse, presence of severe family stressors [1, 14–21] but also after neurosurgery [22–24] and traumatic head injury (THI) [25–28]. It is well known that epileptic seizures are a frequent and well-studied complication of THI [29–31]. However, according to few reports THI may also generate psychiatric symptoms including PNES in both children and adults [7, 25–27].

In this paper we describe a patient with THI who developed paroxysmal behavioral and motor episodes after head trauma (THI), and received a diagnosis of refractory epilepsy for 24 years until she underwent Video-EEG confirming the PNES diagnosis.

## 2. Case Description

A 48-year-old woman was admitted to the Epilepsy Center in July 2005 because of refractory seizures. Seizures began when she was 22 years old, after a bus accident while she was going to work. The bus crashed into a motorcycle, with no loss of awareness. Once outside the bus, she remembered her face was covered with blood and having seen the motorcycle rider being thrown from his motorcycle. After the accident she was admitted at an intensive care unit for a few days and was studied for the THI. During the recovery period it was very difficult for her to walk, and because of continuing lack of sensitivity in both legs she underwent electromyogram (EMG) three months after the accident. The EMG results were normal.

Four months, from the accident seizures began, were characterized by paroxysmal behavioral and motor episodes with a frequency of two to three seizures in a week. She also had absences (lack of response) and sometimes got lost in the street (compatible with dissociative fugue). She received a diagnosis of posttraumatic epilepsy and was treated with antiepileptic drugs like phenytoin, carbamazepine and diazepam. Despite this medication, seizures continued. At this point she was referred to psychotherapy and a psychiatrist prescribed antidepressants for associated depression. That treatment lasted ten years. Because of lack of response to the treatment (the same seizure frequency), many years later she was referred to our Epilepsy Reference Center.

After evaluation by a neurologist she was admitted at the Epilepsy Monitoring Unit in July 2005 and underwent Video-EEG. Neurologic examination was normal, as were an MRI of the brain, interictal SPECT, and interictal electroencephalogram (EEG). Video-EEG monitoring data were obtained with a digital system through 24 hour continuous scalp recordings, with electrodes placed according to the 10–20 international system. The patient was filmed 24 hours a day using a CCD-Panasonic video.

At admission she was taking carbamazepine, phenytoin, and diazepam.

Medication was tapered to discontinuation. Three attacks were recorded during five monitoring days. Video-EEG showed typical episodes characterized by jerking of her trunk at onset, followed by bizarre movements, shaking legs, spitting out, and crying. She complained that it hurt, pointing to her head with her left hand.

These events lasted approximately one minute and she had no total loss of consciousness, with no apparent confusion afterward as she could understand and answer the questions posed by the attending nurse.

These episodes showed no accompanying epileptiform activity on the EEG and because of that, the diagnosis on discharge was PNES. The diagnosis was communicated to the patient and she was referred to us for psychiatric evaluation.

Historical and psychiatric data together with information about social background were obtained, supplemented by information from family and friends. Psychiatric assessment was made by interviews using DSM IV instruments: SCID I Spanish Clinical Version for axis I for psychiatric disorders, SCID II to determine the presence of personality disorders [32, 33], and Hamilton anxiety rating scale [34] for anxiety disorders.

The history of the crash and symptoms triggered including increased arousal, anxiety, nightmares, avoidant symptoms like not taking a bus, and somatic symptoms such as chronic headaches, led to the diagnosis of posttraumatic stress disorder (PTSD) in axis I according to DSM IV [10]. Hamilton anxiety rating scale scored 32 points [34] corresponding to the diagnosis of anxiety disorder. The Structured Clinical Interview for axis II (SCID II) [35] scored for cluster b and c personality disorder [10] with a marked tendency in dependence personality traits.

After psychiatric assessment the patient was told about the emotional origin of her seizures and also that PNESs are often identified in people who suffered traumatic events like hers. Explanation of the PNES diagnosis served as a positive event emphasizing that there was no need to go on antiepileptic drugs was the first terapeutic step. Because of her psychiatric disorder she was prescribed paroxetine, low doses of risperidone and diazepam, and was referred to psychotherapy. A few months later risperidone was tapered off and one and a half years later paroxetine was discontinued. Three years after the evaluation in our center she was diagnosed with panic disorder and was prescribed citalopram 20 mg/d which she remains on at the present time. At present, almost four years after PNES diagnosis, seizure frequency is approximately once a month. She is working now and she is no longer taking antiepileptic drugs.

## 3. Discussion

In the present study we report on a female patient who developed paroxistic episodes after head injury and received the diagnosis of epilepsy for 24 years until PNES diagnosis was confirmed in our epilepsy center with Video-EEG. This is a frequent condition with patients with PNES accounting for 10 to 40 percent of all patients referred for Video-EEG in specialized epilepsy centers [2, 4, 5].

Additionally, between 20 and 40 percent of refractory epileptic patients should be diagnosed with PNES [2, 5, 9, 36]. However when there is a history of THI, the onset of seizures is less suspected to be from psychological origin and is usually thought to be from epileptic origin. Therefore it is usual practice to begin antiepileptic treatment following posthead injury (THI) seizures. This can lead to inadequate results in the PNES cases [7, 28].

PNESs are more frequent in women, who represent around 75% of patients with PNES [6, 37–39] and onset of post-THI PNES also had been described to be more frequent in women [28].

Mean age for seizures onset is nearly 30 years, although they may also appear in childhood or even in elderly people [2, 3, 6, 40]. Delays in PNES diagnoses range from a few months to 9 years [6, 10, 35, 41, 42]. In our working group we found a median duration of PNES previous to diagnosis of 8.70 years [14] and the case patient had been treated without a definitive diagnosis for 24 years.

Several studies in the literature found an association between sexual trauma and PNES [1, 14–22] but less is written about THI and PNES [25–27]. PNESs constitute a problem for neurologist and psychiatrist because differential diagnosis with seizures of epileptic origin sometimes is very difficult especially in patients with THI [7, 28]. Without proper training in the diagnosis of PNES, many of these patients are prescribed antiepileptic drugs that not always beneficial and sometimes are detrimental because they can cause physical side effects, behavioral and cognitive disturbances, and systemic toxicity [10, 40, 43, 44].

In the literature there are a few reports of patients who developed PNES after THI [25–31]. In these cases, the onset of PNES often occurs within first year after head injury, as it is the case of the patient described, but there have been cases described in which PNES may also develop more than 9 years after head trauma [27].

The vulnerability of the brain to behavioral dysfunction after neurologic insult is often cited as a causal pathogenesis model [27]. Many studies correlate the probability of developing seizures with severity of the head injury suggesting that PNES onset is more common after mild THI and epileptic seizures after more serious THI [28, 45–47]. In our case report it seems that the hit on her head was not so serious but the emotional component of the accident was serious enough to cause the psychological reaction which originated PNES.

It has been reported that after diagnosis, in terms of decrease in seizures frequency, half of all patients reduce or stop symptoms, while a quarter becomes chronic [48] while it is significant the decrease in AED use [49].

Despite the ongoing PNES, the patient described above improved her quality of life. She is no longer taking unnecessary drugs and seizure frequency has decreased.

This paper reflects the importance of Video-EEG monitoring and psychiatric assessment among patients with paroxistic disorders resembling epilepsy even in cases where epileptic seizures risk factors such as head trauma injury exist.
